# Association of pre-admission statin use with clinical outcomes in aneurysmal subarachnoid hemorrhage: a multicenter, observational, real-world study

**DOI:** 10.3389/fneur.2026.1828997

**Published:** 2026-04-30

**Authors:** Zhihao Zhao, Shuo Wang, Biao Zhao, Meng Nie, Yunhu Yu, Yang Liu, Yuezheng Dou, Yu Qian, Tao Liu, Rongcai Jiang

**Affiliations:** 1 Department of Neurosurgery, Tianjin Neurological Institute, State Key Laboratory of Experimental Hematology, Laboratory of Post-Neuroinjury Neurorepair and Regeneration in Central Nervous System Tianjin & Ministry of Education, Tianjin Medical University General Hospital, Tianjin, China; 2 Department of Neurosurgery, Xuanwu Hospital, Capital Medical University, Beijing, China; 3 Department of Neurosurgery, The Second Affiliated Hospital of Bengbu Medical University, Bengbu, China; 4 Clinical Research Center for Neurological Disease, The People's Hospital of Honghuagang District of Zunyi, Zunyi, China; 5 Department of Critical Care Medicine, Tianjin Medical University General Hospital, Tianjin, China; 6 The George Institute for Global Health, University of New South Wales, Sydney, NSW, Australia

**Keywords:** aneurysmal subarachnoid hemorrhage, modified Rankin Scale, pre-admission statin, propensity score matching, real-world study

## Abstract

**Background:**

Subarachnoid hemorrhage (SAH) from ruptured intracranial aneurysms is a severe stroke subtype with a high mortality rate. Statins, noted for cholesterol-independent benefits, are being studied as potential treatments, but their effectiveness is debated. This study assesses the impact of pre-admission statin therapy on aneurysmal SAH outcomes.

**Methods:**

This was a multicenter, observational, real-world study from May 2012 to May 2023 at four Chinese tertiary medical centers. Patients were divided into statin and control groups based on pre-admission statin use. The primary outcome was favorable outcome at 30 days, defined as a modified Rankin Scale (mRS) score of 0–2. Propensity score matching (PSM) was applied to balance baseline characteristics. The trial was registered with Chinese Clinical Trial Registry (ChiCTR2300079305).

**Results:**

Out of 1,431 patients, 821 met the inclusion criteria (statin group: 367; control group: 454). After PSM, each group comprised 220 patients. Pre-admission statin was associated with a higher likelihood of favorable outcome at 30 days (before PSM: odds ratio [OR] 3.67, 95% confidence interval [CI] 2.46–5.47, *p* < 0.001; after PSM: OR 5.00, 95% CI 3.00–8.32, *p* < 0.001). Subgroup analysis of the primary outcome revealed significant interactions between pre-admission statin and low-density lipoprotein cholesterol (LDL-C) and alcohol consumption (Both P for interaction < 0.05). The benefit of statin at 30 days was more pronounced in patients with LDL-C < 3.4 mmol/L and non-drinkers.

**Conclusion:**

Pre-admission statin therapy was associated with improved prognosis at 30 days in patients with aneurysmal SAH, however this benefit could not extend to 90 days. The effect of statin appeared to vary according to baseline LDL-C levels and alcohol consumption. Further multicenter, international randomized controlled trials are warranted to confirm these findings.

## Introduction

Subarachnoid hemorrhage (SAH) is a rare and severe subtype of stroke, with a global incidence rate of approximately 9 per 100,000 person-years, accounting for approximately 5% of all strokes (1). Despite recent and notable advancements in treatment over recent decades, SAH still carries a high median case fatality rate, ranging from 27 to 44% across diverse regions worldwide (2). The primary cause of spontaneous SAH is the rupture of intracranial aneurysms, which accounts for around 85% of cases. This typically occurs in younger patients, resulting in a significant loss of life expectancy comparable to other more common stroke types (3). While endovascular coiling and surgical clipping are effective interventions for occluding these dilated aneurysms, survivors remain vulnerable to secondary complications, such as rebleeding, hydrocephalus, seizures, and delayed cerebral ischemia (DCI) related to cerebral vasospasm (CVS) (4).

At present, nimodipine stands as the sole recommended pharmacological intervention (Class I; Level of Evidence A) for immediate SAH management, believed to mitigate DCI and unfavorable outcomes in SAH patients (5). The exploration of more effective pharmaceutical interventions to improve the prognosis of SAH has been a focus of researchers. Statins, known for their multifaceted cholesterol-independent benefits (e.g., anti-inflammatory and antioxidant effects) and favorable safety profile (6–8), have long been considered candidate agents for improving SAH outcomes (9). Earlier retrospective studies hinted at the considerable potential of statins to enhance outcomes in SAH patients (10). However, meta-analyses did not support the notion that statins reduce mortality and mitigate delayed neurological deficits. Nevertheless, they did indicate some effectiveness in improving CVS (11, 12). CVS typically occurs 3–4 days after SAH, peaking at 7–10 days; hence, the early administration of statins may hold promise in in enhancing outcomes by alleviating CVS. In a recent retrospective study, long-term statin use prior to aSAH was associated with a significant decrease in symptomatic vasospasm (13). However, existing studies on pre-admission statin use often encompassed limited sample sizes in the statin treatment groups and lacked well-controlled data (13–16).

Currently, with statins being included in primary and secondary prevention for various chronic diseases, there has been a substantial rise in the proportion of the population using statins, accompanied by shifts in the types of statins being employed (17, 18). This may alter the impact of statins on aneurysmal SAH patients, necessitating a reassessment. Therefore, we conducted a multi-center, propensity score-matched study to investigate whether pre-admission statin use could affect the prognosis of patients with aneurysmal SAH and identify possible contributing factors.

## Methods

### Study design and patient selection

This hospital-based, multi-center, observational, real-world cohort study of consecutive patients with aneurysmal SAH was conducted at four tertiary medical centers in China between May 2012 and May 2023. This study adhered to the ethical standards outlined in the 1964 Declaration of Helsinki and its subsequent amendments. The Ethics Committee of Tianjin Medical University General Hospital approved the study (Approval No. IRB2025-YX-025-01). All patients were provided with written informed consent. This study was registered in the Chinese Clinical Trial Center (registration number ChiCTR2300079305). Our study adhered to the STROBE (Strengthening the Reporting of Observational Studies in Epidemiology) statement and its checklist guidelines (19).

The inclusion criteria were as follows: (1) both male and female adults aged over 18 years; (2) present with acute SAH diagnosed by computed tomography (CT); (3) confirmation of an intracranial aneurysm by digital subtraction angiography, CT angiography, or magnetic resonance angiography; (4) admitted within 72 h following SAH; (5) Surgical options for aneurysms (clipping or coiling) initiated within 48 h of admission; (6) absence of other significant intracranial diseases, including but not limited to pre-admission diagnoses of Moyamoya disease, multiple sclerosis, dementia, epilepsy disorder, cerebral infarction, encephalitis, or malignant brain tumor; (7) availability of complete clinical data (the first required laboratory results after onset are obtained within 72 h, and no fewer than two reexaminations are performed during hospitalization); and (8) actively accept symptom-guided basic symptomatic treatment and willing to participate in follow-up. Conversely, the main exclusion criteria were: (1) SAH caused by trauma and other non-aneurysm rupture; (2) a history of neurological functional score-affecting diseases (including organic or severe psychiatric disorders such as schizophrenia, depression with somatization affecting daily life, autism, etc.); (3) concurrent serious systemic diseases including systemic infection, multiple organ failure, and gastrointestinal bleeding; (4) insufficient or indistinct required laboratory data and reexaminations; (5) recent use of glucocorticoids; (6) concurrent liver dysfunction; (7) extremely prolonged or abbreviated hospital stays (<3 or >80 days); and (8) patients declining follow-up for various reasons or demonstrating poor cooperation during follow-up assessments.

### Data collection and outcome measures

Two independent researchers were trained in advance to establish consistent standards for inclusion and exclusion criteria and to conduct the initial selection of patients. Clinical data were recorded using electronic standardized forms, with any discrepancies being resolved through discussion. We retrieved information from electronic medical records, including baseline demographic characteristics, severity of illness at admission, prior medical history, laboratory findings, treatment information and adverse events during hospitalization. Patients were categorized into statin and control groups depending on whether they had undergone statin therapy for a duration exceeding 2 weeks in the month preceding the onset of SAH.

At 30 days and 90 days after SAH, we evaluated the neurological status of patients and classified their functional prognosis according to the modified Rankin Scale (mRS) score, which was categorized into six grades: no symptoms at all (grade 0), no significant disability despite symptoms (grade 1), slight disability (grade 2), moderate disability (grade 3), moderately severe disability (grade 4), severe disability (grade 5), and death (grade 6). Researchers conducted telephonic follow-ups separately, contacting the subject themselves or their guardian, and resolved any discrepancies through negotiation. Transaminase elevation and neutrophil elevation were defined based on local reference range, with the presence of one or more readings above the upper limit during hospitalization considered positive.

The primary outcome was favorable outcome at 30 days, defined as an mRS score of 0–2. The selection of secondary outcomes was predetermined to offer corroborative evidence concerning the primary outcome. Secondary outcomes encompassed favorable outcome and all-cause mortality at 90 days after SAH, recurrent cerebral hemorrhage, hydrocephalus, external ventricular drainage, heart failure, seizure, neutrophil elevation and transaminase elevation during hospitalization, and length of hospital stay.

### Statistical analysis

Continuous variables were summarized using mean and standard deviation (SD) and analyzed with Student *t*-test or Mann–Whitney U test after normality assessment. Categorical variables underwent Chi-square or Fisher’s exact tests.

Propensity score matching (PSM) controlled for confounding, with propensity scores derived from logistic regression on baseline variables. In detail, logistic regression was utilized to establish a propensity score. This score considered variables identified by significant differences in baseline characteristics within the unmatched cohort, as well as variables deemed relevant to observed outcomes. Ultimately, the score comprised age, gender, systolic blood pressure, glucose, LDL-C levels, medical history of hypertension, diabetes, heart disease and stroke, anticoagulant use, smoking, drinking, and WFNS grade. Subjects were 1:1 matched using nearest neighbor matching, with a 0.2 SD caliper for the logit of propensity scores, without replacement. Covariate balance was assessed using standardized mean differences (SMD), with values above 10% indicating meaningful imbalances.

Multivariable logistic regression and linear regression were employed to estimate odds ratios (ORs) and mean differences (MDs), respectively, each presented with 95% confidence intervals (CIs), for categorical and continuous variables. The covariates adjusted for were consistent with those in the PSM analysis. The robustness of the observed associations against residual confounding was quantified through the calculation of E-values (20). Subgroup analyses were conducted to examine effect heterogeneity on the primary outcome according to age, sex, SBP, LDL-C, smoking, alcohol consumption, and WFNS grade. The LDL-C threshold of 3.4 mmol/L was chosen based on the guidelines for the management of dyslipidaemias, which define LDL-C ≥ 3.4 mmol/L (≥130 mg/dL) as moderately elevated. Missing data were imputed using the missForest algorithm (21, 22).

Significance was set at two-sided *p* values <0.05. Data analysis was conducted using R version 4.5.1.

## Results

### Patient characteristics

**Figure 1:**
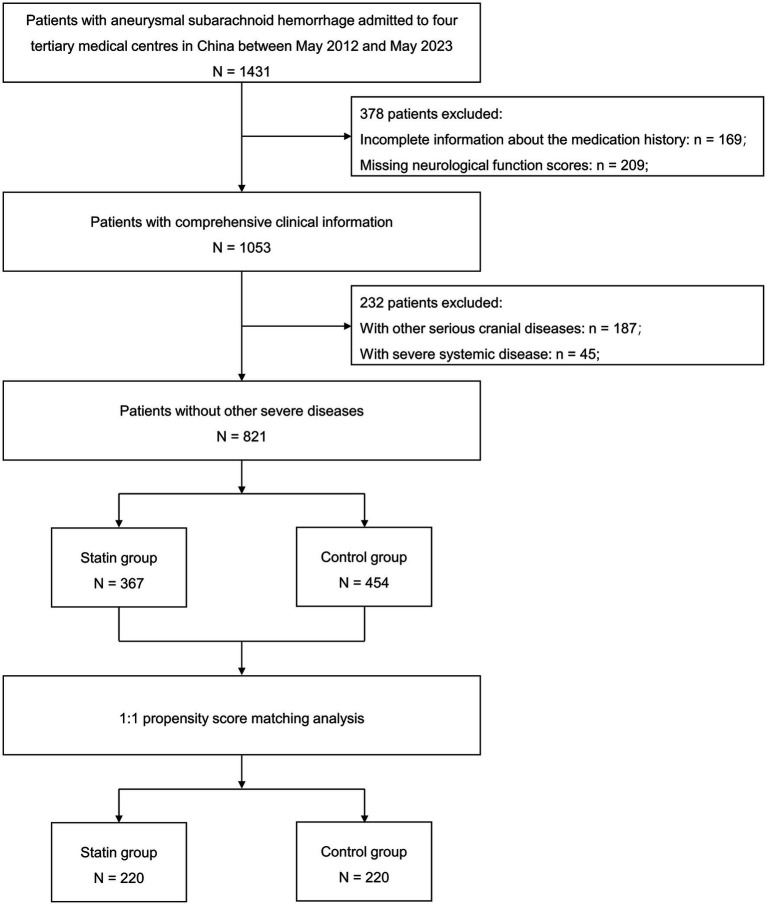
Flow chart detailing the patient selection process for the study.

Between May 2012 and May 2023, a total of 1,431 individuals were admitted due to aneurysmal SAH. After evaluation, 821 patients were ultimately included in this study (Figure 1). Out of these patients, 367 (44.7%) had a recorded history of statin usage before their admission, while the remaining 454 (55.3%) reported no such prior use. With a threshold of SMD > 0.1 for meaningful imbalances, baseline characteristics exhibited differences in age, LDL-C concentration, medical histories (including hypertension, diabetes, heart disease and stroke), prior anticoagulant usage, alcohol consumption, and the WFNS grade. The statin group exhibited higher mean age (59.55 ± 12.95 vs. 66.67 ± 12.98 years, SMD = 0.549), elevated LDL-C levels (2.75 ± 0.92 vs. 2.98 ± 0.73 mmol/L, SMD = 0.275), increased rates of diabetes (17.0% vs. 29.2%, SMD = 0.293), heart disease (5.1% vs. 41.4%, SMD = 0.953), anticoagulant medication usage (5.3% vs. 12.8%, SMD = 0.264), and higher WFNS grades (2 [1–4] vs. 3 [2–4], SMD = 0.326). Following PSM, 220 patients with pre-admission statin use were successfully matched with 220 patients who did not use statins. This matching process resulted in excellent balance among all baseline characteristics (Table 1). The distribution of missing data is detailed in Supplementary Table 1.

**Table 1:** Baseline characteristics of the unmatched and matched participants.

Characteristic	Before PSM				After PSM			
	Overall (*n* = 821)	Control group (*n* = 454)	Statin group (*n* = 367)	SMD^a^	Overall (*n* = 440)	Control group (*n* = 220)	Statin group (*n* = 220)	SMD^a^
Age, mean ± SD, years	62.74 ± 13.43	59.55 ± 12.95	66.67 ± 12.98	0.549	64.91 ± 12.16	64.71 ± 12.03	65.11 ± 12.32	0.031
Gender, male (%)	496 (60.4)	273 (60.1)	223 (60.8)	0.013	273 (62.0)	139 (63.2)	134 (60.9)	0.047
Systolic blood pressure, mean ± SD, mmHg	166.98 ± 26.91	165.64 ± 26.92	168.62 ± 26.85	0.111	166.94 ± 27.19	167.61 ± 28.30	166.27 ± 26.09	0.050
Diastolic blood pressure, mean ± SD, mmHg	94.04 ± 13.50	93.81 ± 14.33	94.33 ± 12.41	0.039	92.58 ± 13.62	92.58 ± 14.31	92.59 ± 12.92	0.001
Glucose, mean ± SD, mmol/L	7.82 ± 2.98	7.69 ± 3.19	7.99 ± 2.69	0.102	7.69 ± 2.52	7.80 ± 2.74	7.58 ± 2.28	0.079
LDL-C, mean ± SD, mmol/L	2.86 ± 0.84	2.75 ± 0.92	2.98 ± 0.73	0.275	2.98 ± 0.78	2.97 ± 0.85	2.98 ± 0.70	0.024
Smoking, *n* (%)	150 (18.3)	90 (19.8)	60 (16.3)	0.090	61 (13.9)	30 (13.6)	31 (14.1)	0.012
Alcohol consumption, *n* (%)	112 (13.6)	74 (16.3)	38 (10.4)	0.176	34 (7.7)	18 (8.2)	16 (7.3)	0.030
Medical history, *n* (%)								
Hypertension	524 (63.8)	304 (67.0)	220 (59.9)	0.146	268 (60.9)	130 (59.1)	138 (62.7)	0.074
Diabetes	184 (22.4)	77 (17.0)	107 (29.2)	0.293	110 (25.0)	52 (23.6)	58 (26.4)	0.060
Heart disease	175 (21.3)	23 (5.1)	152 (41.4)	0.953	56 (12.7)	23 (10.5)	33 (15.0)	0.092
Stroke	86 (10.5)	56 (12.3)	30 (8.2)	0.137	46 (10.5)	23 (10.5)	23 (10.5)	<0.001
Prior use of antithrombotic agents	71 (8.6)	24 (5.3)	47 (12.8)	0.264	41 (9.3)	19 (8.6)	22 (10.0)	0.041
WFNS grade, median [IQR]	3 [1–4]	2 [1–4]	3 [2–4]	0.326		2 [1–4]	3 [1–4]	0.006
I, *n* (%)	238 (29.0)	154 (33.9)	84 (22.9)		125 (28.4)	60 (27.3)	65 (29.5)	
II, *n* (%)	170 (20.7)	97 (21.4)	73 (19.9)		94 (21.4)	51 (23.2)	43 (19.5)	
III, *n* (%)	152 (18.5)	80 (17.6)	72 (19.6)		84 (19.1)	41 (18.6)	43 (19.5)	
IV, *n* (%)	164 (20.0)	86 (18.9)	78 (21.3)		88 (20.0)	45 (20.5)	43 (19.5)	
V, *n* (%)	97 (11.8)	37 (8.1)	60 (16.3)		49 (11.1)	23 (10.5)	26 (11.8)	
Treatment, *n* (%)				0.080				0.082
Clipping	335 (40.8)	178 (39.2)	157 (42.8)		180 (40.9)	89 (40.5)	91 (41.4)	
Coiling	337 (41.0)	189 (41.7)	148 (40.3)		175 (39.8)	85 (38.6)	90 (40.9)	
Others	149 (18.1)	87 (19.2)	62 (16.9)		85 (19.3)	46 (20.9)	39 (17.7)	

IQR, interquartile range; LDL-C, low-density lipoprotein cholesterol; SD, standard deviation; SMD, standard mean difference; WFNS, World Federation of Neurosurgical Societies.

a SMD >0.1 indicates imbalance.

### Outcomes

**Figure 2:**
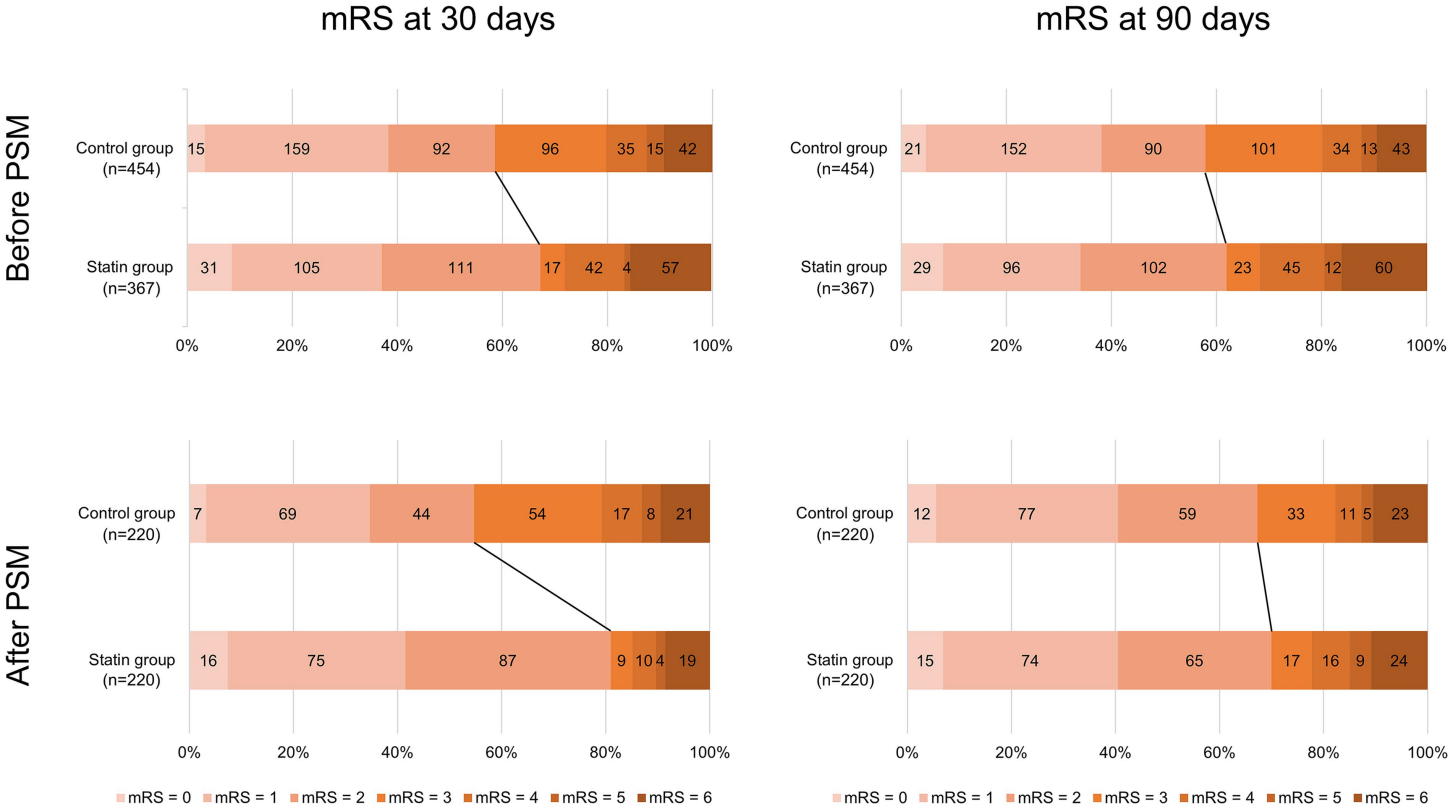
The shift in the modified Rankin Scale (mRS) scores at 30 days and 90 days after SAH of the control group and the statin group.

In both unmatched and matched cohort, pre-admission statin was associated with a higher likelihood of favorable outcome at 30 days (before PSM: OR 3.67, 95% CI 2.46–5.47, *p* < 0.01; after PSM: OR 5.00, 95% CI 3.00–8.32, *p* < 0.001; Table 2). However, although a trend towards improved prognosis at 90 days was observed, this did not reach statistical significance (before PSM: OR 1.18, 95% CI 0.84–1.67, *p* = 0.255; after PSM: OR 1.44, 95% CI 0.94–2.23, *p* = 0.096; Table 2). The shift in the mRS scores at 30 days and after 90 days is depicted in Figure 2.

**Table 2:** Association of statin therapy (statin non-user as reference) with primary and secondary outcomes.

Variables	Before PSM analysis				After PSM analysis			
	Control group (*n* = 454)	Statin group (*n* = 367)	OR/MD (95%CI)^a^	*P* value	Control group (*n* = 220)	Statin group (*n* = 220)	OR/MD (95%CI)^a^	*P* value
Primary outcome								
Favorable outcome at 30 days, *n* (%)^b^	266 (58.6)	247 (67.3)	3.67 (2.46–5.47)	<0.001	120 (54.5)	178 (80.9)	5.00 (3.00–8.32)	<0.001
Secondary outcome								
Favorable outcome at 90 days, *n* (%)^b^	263 (57.9)	227 (61.9)	1.18 (0.84–1.67)	0.255	148 (67.3)	166 (75.5)	1.44 (0.94–2.23)	0.096
All-cause mortality at 90 days, *n* (%)	42 (9.3)	57 (15.5)	0.87 (0.47–1.61)	0.646	21 (9.5)	19 (8.6)	0.70 (0.31–1.59)	0.392
Recurrent cerebral hemorrhage, *n* (%)	22 (4.8)	23 (6.3)	1.86 (0.79–4.56)	0.176	6 (2.7)	10 (4.5)	1.52 (0.46–5.07)	0.492
Hydrocephalus, *n* (%)	166 (36.6)	108 (29.4)	0.68 (0.47–0.98)	0.040	73 (33.2)	58 (26.4)	0.75 (0.49–1.16)	0.199
External ventricular drainage, *n* (%)	85 (18.7)	33 (9.0)	0.38 (0.22–0.67)	0.001	37 (16.8)	13 (5.9)	0.26 (0.12–0.54)	<0.001
Seizure, *n* (%)	9 (2.0)	22 (6.0)	6.53 (0.88–48.34)	0.066	4 (1.8)	2 (0.9)	0.84 (0.12–6.05)	0.864
Neutrophil elevation, *n* (%)	168 (37.0)	206 (56.1)	1.65 (1.12–2.42)	0.011	85 (38.6)	115 (52.3)	1.72 (1.09–2.97)	0.019
Transaminase elevation, *n* (%)	30 (6.6)	85 (23.2)	3.01 (1.77–5.14)	<0.001	20 (9.1)	46 (20.9)	3.44 (1.82–6.50)	<0.001
Hospital stay, mean ± SD, days	19.42 ± 14.28	23.19 ± 18.33	2.85 (0.19–5.52)	0.036	18.86 ± 14.14	23.21 ± 17.66	3.73 (0.84, 6.62)	0.005

OR, odds ratio; MD, mean difference; CI, confidence interval.

a Adjusted for age, gender, systolic blood pressure, glucose, LDL, smoking, alcohol consumption, medical histories of hypertension, diabetes, heart disease, and stroke, prior use of antithrombotic agents, and WFNS grade. Effect sizes for all categorical variables are presented as ORs with their 95% CIs, while the continuous variable Hospital stay is expressed as MD with its 95% CI.

b Favorable outcome was defined as a mRS score of 0–2.

For secondary outcomes, pre-admission statin therapy was associated with a reduced requirement for external ventricular drainage (OR 0.26, 95% CI 0.12–0.54, *p* < 0.001). Conversely, statin use was associated with a higher risk of neutrophil elevation (OR 1.72, 95% CI 1.09–2.97, *p* = 0.019), transaminase elevation (OR 3.44, 95% CI 1.82–6.05, *p* < 0.001), and a longer length of hospital stay (MD 3.73 days, 95% CI 0.84–6.62, *p* = 0.005) (Table 2). No significant differences were observed between the two groups in terms of all-cause mortality, recurrent cerebral hemorrhage, hydrocephalus, or seizures. Univariable analyses in both unmatched and matched cohorts yielded results consistent with the multivariable analyses (Supplementary Tables 2, 3). In addition, E-value analyses for all primary and secondary outcomes indicated that the observed associations were robust to potential unmeasured confounding (Supplementary Table 4).

### Subgroup analysis

**Figure 3:**
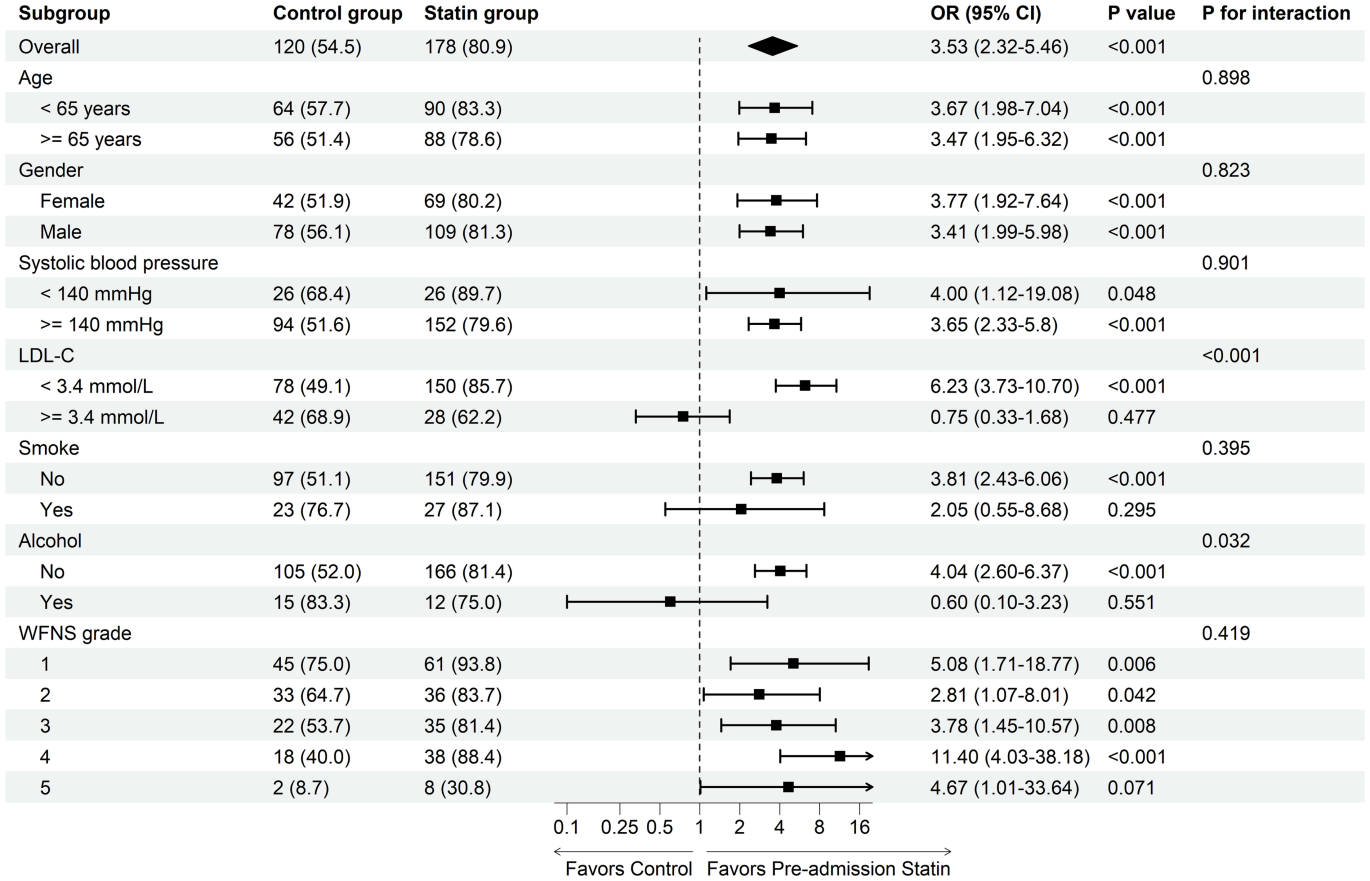
Subgroup analysis of the primary outcome in the matched cohort.

Subgroup analysis of the primary outcome demonstrated significant interactions between pre-admission statin use and both LDL-C level (P for interaction <0.001) and alcohol consumption (P for interaction = 0.032) (Figure 3). Statin therapy was associated with a higher likelihood of favorable outcome at 30 days in patients with LDL-C < 3.4 mmol/L (OR 6.23, 95% CI 3.73–10.70, *p* < 0.001), but not in those with LDL-C ≥ 3.4 mmol/L (OR 0.75, 95% CI 0.33–1.68, *p* = 0.477). Similarly, a benefit was observed in non-drinkers (OR 4.04, 95% CI 2.60–6.37, *p* < 0.001) but not in drinkers (OR 0.60, 95% CI 0.10–3.23, *p* = 0.551).

## Discussion

In this multi-center, observational, real-world study, our matching design efficiently balanced the baseline and treatment differences, resulting in a 1:1 match of 440 the enrolled patients with aneurysmal SAH, with or without pre-admission statin use. Our results showed that pre-admission statin use was associated with improved favorable outcome at 30 days. The increase in OR from 3.67 to 5.00 after PSM may be explained by more effective control of confounding variables through propensity score matching, which reduces bias and yields a less confounded estimate of the treatment effect. Previous research primarily focused on whether statin therapy provided additional protection when aneurysms ruptured. The STASH trial, the most well-known in assessing statin treatment for aneurysmal SAH patients, found that 40 mg of daily simvastatin did not improve mRS scores at discharge or 6-month follow-up, nor reduce patient mortality (23). Subsequent randomized controlled trials (RCTs) similarly reported that administering higher doses of simvastatin (80 mg daily) did not yield better outcomes (24). However, both of these studies commenced statin treatment within 96 h of diagnosis and maintained it for 2–3 weeks. Questions regarding the appropriateness of this time window and the potential for extended statin therapy to confer greater benefits are worth exploring, as other researchers have expressed concerns. These revolve around the possibility of false negatives being observed in 2–3 weeks course of treatment, suggesting the need for longer-term statins treatment (25), or some negative rebound effect (26).

Additionally, we observed that pre-admission statin use was associated with a decreased requirement for external ventricular drainage, potentially due to a lower (albeit statistically non-significant) incidence of hydrocephalus in the statin group compared to controls. The rupture of aneurysm frequently results in intraventricular hemorrhage, closely linked to the development of hydrocephalus and serving as an independent predictor of poor prognosis (27, 28). After SAH, blood and degradation products from red blood cells, such as hemoglobin, hemiglobin, and iron ions, diffuse into the ventricular system and subarachnoid space. On one hand, these substances deposit and interfere with the normal absorption and circulation of cerebrospinal fluid, leading to the formation of hydrocephalus. On the other hand, these substances early disrupt the balance between the production and scavenging of oxygen-derived free radicals, damage the blood–brain barrier, activate immune cells (such as astrocytes and microglia), trigger inflammatory responses, exacerbate brain ischemia and edema following SAH, increasing the risk of secondary bleeding (29). Experimental studies in rodents have extensively demonstrated the anti-inflammatory, antioxidative stress, and endothelial function-improving effects of statins independent of cholesterol reduction, which may be crucial in reducing adverse events in SAH patients (30, 31). Wang et al. demonstrated that simvastatin could enhance the phagocytosis of red blood cells by microglia, thereby promoting hematoma absorption, alleviating hydrocephalus, and improving neurological recovery in experimental cerebral hemorrhage animals (32, 33). Rudolph et al. reported a long-term follow-up case–control study involving 16,235 patients with intracranial hemorrhage, revealing that the longer the duration of statin use, the lower the risk of intracranial hemorrhage (34). These findings, along with our discoveries, suggest that pre-admission statin use may benefit SAH patients via multiple biological mechanisms, thereby pointing to a potential for improved prognosis.

However, 90 days post-SAH, there was no significant difference in favorable outcome between the statin and control groups. This finding may suggest that the putative neuroprotective effects of statins in the acute phase of SAH do not necessarily confer a lasting advantage on long-term neurological recovery. A study that evaluates the pre-admission, post-admission and post-discharge statin use of patients with SAH can provide valuable insights into the dynamics of the mRS. A recently published study on aneurysmal SAH revealed that patients with pre-ictal and continued use of statins, primarily Atorvastatin (constituting 53.2%), demonstrated similar mRS and Glasgow outcome scale extended (GOSE) scores compared to non-statin users (median follow-up time of 5.3 months). In the matched pair analysis, which balanced differences in age, gender, and hypertension, statin users showed a significantly higher proportion of GOSE 6–8 and an increasing trend in mRS 0–2 (*p* = 0.078) (35). Another RCT involving elderly Chinese patients with aneurysmal SAH indicated that, after aneurysm clipping or embolization, patients using atorvastatin for 2 weeks exhibited a slight increasing trend in GOS 4–5 at month 6 (83.3% vs. 87.3%), although the difference was not statistically significant (36). Presently, it is suggested that maintaining statin therapy in individuals already using statins before the onset of cerebral hemorrhage improves patient outcomes (37). Nevertheless, a longer follow-up period and a more meticulous control of the timing and duration of statin use are essential to assess any additional benefits of statins beyond their potential to accelerate recovery from the acute phase of aneurysmal SAH.

The safety of pre-admission statin therapy for aneurysmal SAH patients lacks sufficient reporting. Our study showed no significant difference in mortality between the groups, rendering a worst-case scenario analysis futile. Nonetheless, we did observe a higher incidence of transaminase elevation during hospitalization in the statin group. However, no distinct statin-related adverse events severe enough to warrant discontinuation were recorded, such as impaired liver function, myopathy (companied by creatine kinase concentration exceeding 10 times the upper limit of normal), new-onset diabetes mellitus, or other rarer adverse events. While statins are recognized for their hepatotoxic effects, they seldom cause clinical symptoms or irreversible organ failure (38, 39). Some research suggests that statin-induced hepatotoxicity may be primarily related to the specific type and dosage of statin used, rather than LDL-C reduction (39), underscoring the importance of considering these factors in clinical practice. However, our study had constraints in assessing statin tolerability in patients with aneurysmal SAH. The inclusion criteria for “pre-admission use of statins” may have excluded some patients who could not tolerate statins early on. Additionally, mild symptomatic adverse events that may be statin-related, such as headache, nausea, dizziness, diarrhea, constipation, and musculoskeletal discomfort, may have been masked by more obvious symptoms associated with aneurysmal SAH. Furthermore, confounding factors like statin dose and drug–drug interactions made evaluation challenging. Nevertheless, based on international consensus and the outcome indicators we have gathered, the probability of severe consequences from statin use in aneurysmal SAH patients is exceedingly low. Common symptomatic adverse reactions are generally manageable, providing an overall indication of safety (40, 41).

A meta-analysis that exclusively included RCTs indicated that statin therapy for aneurysmal SAH patients might increase the risk of bacteremia while not seizures (42), consistent with our results, which revealed a significant neutrophil elevation in the statin group but no difference in the occurrence of seizures. Given the absence of additional infection indicators, it remained inconclusive whether the neutrophil elevation suggested an increased risk of local infection or bacteremia.

Subgroup analysis of the primary outcome revealed significant interactions between pre-admission statin use and both LDL-C level and alcohol consumption. Statin therapy was associated with a higher likelihood of favorable outcome at 30 days in patients with LDL-C < 3.4 mmol/L and non-drinkers, but not in those with LDL-C ≥ 3.4 mmol/L or drinkers. Elevated LDL-C and alcohol consumption are known to be associated with increased cardiovascular risk (43, 44). This hints that the unhealthy cardiac status in in patients with aneurysmal SAH may diminish the potential benefits of pre-admission statin therapy. Moreover, chronic alcohol consumption exacerbates systemic and cerebrovascular inflammatory responses and oxidative stress (45, 46), which may thereby attenuate the protective effects of statins conferred through their anti-inflammatory and vascular-stabilizing properties.

Although our study initially explores the feasibility of pre-admission statin therapy for patients with unruptured aneurysms, there are some limitations. First, given the observational nature of this real-world study, treatment allocation to the statin and control groups was not randomized, and residual confounding could not be entirely excluded. To minimize bias, we performed propensity score matching and multivariable regression analyses to adjust for measured confounders, while unadjusted models were also presented as sensitivity analyses. In addition, E-value analyses for all outcomes suggested that the observed associations were robust and unlikely to be fully attributable to unmeasured confounding. Additionally, as this was an observational study, the available variables were limited to those routinely recorded in clinical practice. Consequently, several cranial outcomes among patients with aneurysmal SAH, such as cerebral vasospasm CVS and DCI, were not available. Nevertheless, we assessed functional outcomes at multiple time points and reported the incidence of relevant adverse events, which together provide supportive evidence regarding the efficacy and safety of pre-admission statin therapy. Finally, our study population consisted exclusively of Chinese patients. Although this provides valuable evidence for Asian populations, it may limit the generalizability of our findings to other ethnic groups. Future multicenter, international RCTs are warranted to further validate these findings and provide more robust evidence.

## Conclusion

Pre-admission statin use was associated with improved 30-day outcomes in patients with aneurysmal subarachnoid hemorrhage, but not with outcomes at 90 days. This association appeared to differ according to baseline LDL-C levels and alcohol consumption, with greater benefit observed in patients with LDL-C levels below 3.4 mmol/L and in non-drinkers. These findings require further validation through multicenter, international RCTs.

## Data Availability

The raw data supporting the conclusions of this article will be made available by the authors, without undue reservation.
