# Supplementary material for: Association of pre-admission statin use with clinical outcomes in aneurysmal subarachnoid hemorrhage: a multicenter, observational, real-world study
Source: Front Neurol. 2026 Apr 30;17:1828997. doi: 10.3389/fneur.2026.1828997 (PMC13171303; doi:10.3389/fneur.2026.1828997)
Supplement: Supplementary file 1 [file Table_1.DOCX]

**Supplementary materials**

**(Association of Pre-admission Statin Use With Clinical Outcomes in Aneurysmal Subarachnoid Hemorrhage: A Multicenter, Observational, Real-world Study)**

**Contents**

[eTable 1. Missing Data by Study Group 2](#_Toc223859950)

[eTable 2. Univariate analysis of the unmatched participants 3](#_Toc223859951)

[eTable 3. Univariate analysis of the matched participants 4](#_Toc223859952)

[eTable 4. E-value for the effect of Pre-admission statin on the primary and secondary outcomes 5](#_Toc223859953)

**eTable 1. Missing Data by Study Group**

| **Variable*** | **Overall**  **(n=821)** | **Control group**  **(n=454)** | **Statin group**  **(n=367)** | ***P* value** |
| --- | --- | --- | --- | --- |
| Age | 8 (1.0) | 5 (1.1) | 3 (0.8) | 0.681 |
| Systolic blood pressure | 28 (3.4) | 12 (2.6) | 16 (4.4) | 0.178 |
| Diastolic blood pressure | 32 (3.9) | 15 (3.3) | 17 (4.6) | 0.328 |
| Glucose | 33 (4.0) | 18 (4.0) | 15 (4.1) | 0.929 |
| LDL-C | 38 (4.6) | 21 (4.6) | 17 (4.6) | 0.996 |
| Hypertension | 6 (0.7) | 4 (0.9) | 2 (0.5) | 0.574 |
| Diabetes | 8 (1.0) | 3 (0.7) | 5 (1.4) | 0.309 |
| Heart disease | 9 (1.1) | 3 (0.7) | 6 (1.6) | 0.183 |
| Stroke | 3 (0.4) | 2 (0.4) | 1 (0.3) | 0.692 |
| Prior use of antithrombotic agents | 26 (3.2) | 13 (2.9) | 13 (3.5) | 0.581 |
| Smoking | 18 (2.2) | 9 (2.0) | 9 (2.5) | 0.648 |
| Alcohol consumption | 22 (2.7) | 11 (2.4) | 11 (3.0) | 0.612 |
| Favorable outcome at 30 days | 12 (1.5) | 5 (1.1) | 7 (1.9) | 0.339 |
| Favorable outcome at 90 days | 25 (3.0) | 15 (3.3) | 10 (2.7) | 0.631 |
| All-cause mortality at 90 days | 25 (3.0) | 15 (3.3) | 10 (2.7) | 0.631 |
| Recurrent cerebral hemorrhage | 12 (1.5) | 5 (1.1) | 7 (1.9) | 0.339 |
| Hydrocephalus | 17 (2.1) | 12 (2.6) | 5 (1.4) | 0.200 |
| External ventricular drainage | 10 (1.2) | 6 (1.3) | 4 (1.1) | 0.764 |
| Seizure | 11 (1.3) | 7 (1.5) | 4 (1.1) | 0.575 |
| Neutrophil elevation | 9 (1.1) | 7 (1.5) | 2 (0.5) | 0.173 |
| Transaminase elevation | 21 (2.6) | 12 (2.6) | 9 (2.5) | 0.863 |
| Hospital stay | 25 (3.0) | 12 (2.6) | 13 (3.5) | 0.456 |

* Missing data were presented as n (%).

**eTable 2. Univariate analysis of the unmatched participants**

| **Variables** | **Total (n=821)** | **Control group (n=454)** | **Statin group (n=367)** | **Univariate analysis** | |
| --- | --- | --- | --- | --- | --- |
|  |  |  |  | **OR/MD (95%CI) ^a^** | **P value** |
| **Primary outcome** |  |  |  |  |  |
| Favorable outcome at 30 days, n (%) ^b^ | 513 (62.5) | 266 (58.6) | 247 (67.3) | 1.46 (1.09-1.94) | 0.011 |
| **Secondary outcome** |  |  |  |  |  |
| Favorable outcome at 90 days, n (%) ^b^ | 490 (59.7) | 263 (57.9) | 227 (61.9) | 1.18 (0.89-1.56) | 0.255 |
| All-cause mortality at 90 days, n (%) | 99 (12.1) | 42 (9.3) | 57 (15.5) | 1.80 (1.18-2.76) | 0.007 |
| Recurrent cerebral hemorrhage, n (%) | 45 (5.5) | 22 (4.8) | 23 (6.3) | 1.31 (0.72-2.40) | 0.375 |
| Hydrocephalus, n (%) | 274 (33.4) | 166 (36.6) | 108 (29.4) | 0.73 (0.54-0.97) | 0.031 |
| External ventricular drainage, n (%) | 118 (14.4) | 85 (18.7) | 33 (9.0) | 0.43 (0.28-0.66) | <0.001 |
| Seizure, n (%) | 31 (3.8) | 9 (2.0) | 22 (6.0) | 3.16 (1.43-6.94) | 0.004 |
| Neutrophil elevation, n (%) | 374 (45.6) | 168 (37.0) | 206 (56.1) | 2.18 (1.65-2.88) | <0.001 |
| Transaminase elevation, n (%) | 115 (14.0) | 30 (6.6) | 85 (23.2) | 4.26 (2.74-6.63) | <0.001 |
| Hospital stay, mean ± SD, days | 21.10 ± 16.31 | 19.42 ± 14.28 | 23.19 ± 18.33 | 3.77 (1.54, 6.00) | <0.001 |

**Abbreviations:** OR, odds ratio; MD, mean difference; CI, confidence interval.

^a^ Effect sizes for all categorical variables are presented as ORs with their 95% CIs, while the continuous variable Hospital stay is expressed as MD with its 95% CI.

^b^ Favorable outcome was defined as a mRS score of 0-2.

**eTable 3. Univariate analysis of the matched participants**

| **Variables** | **Total (n=440)** | **Control group (n=220)** | **Statin group (n=220)** | **Univariate analysis** | |
| --- | --- | --- | --- | --- | --- |
|  |  |  |  | **OR/MD (95%CI) ^a^** | **P value** |
| **Primary outcome** |  |  |  |  |  |
| Favorable outcome at 30 days, n (%) ^b^ | 298 (67.7) | 120 (54.5) | 178 (80.9) | 3.53 (2.32-5.46) | <0.001 |
| **Secondary outcome** |  |  |  |  |  |
| Favorable outcome at 90 days, n (%) ^b^ | 314 (71.4) | 148 (67.3) | 166 (75.5) | 1.50 (0.99-2.27) | 0.058 |
| All-cause mortality at 90 days, n (%) | 40 (9.1) | 21 (9.5) | 19 (8.6) | 0.90 (0.46-1.72) | 0.740 |
| Recurrent cerebral hemorrhage, n (%) | 16 (3.6) | 6 (2.7) | 10 (4.5) | 1.69 (0.62-5.07) | 0.313 |
| Hydrocephalus, n (%) | 131 (29.8) | 73 (33.2) | 58 (26.4) | 0.72 (0.48-1.09) | 0.118 |
| External ventricular drainage, n (%) | 50 (11.4) | 37 (16.8) | 13 (5.9) | 0.31 (0.15-0.59) | <0.001 |
| Seizure, n (%) | 6 (1.4) | 4 (1.8) | 2 (0.9) | 0.49 (0.07-2.57) | 0.420 |
| Neutrophil elevation, n (%) | 200 (45.5) | 85 (38.6) | 115 (52.3) | 1.73 (1.19-2.55) | 0.004 |
| Transaminase elevation, n (%) | 66 (15.0) | 20 (9.1) | 46 (20.9) | 2.64 (1.53-4.73) | <0.001 |
| Hospital stay, mean ± SD, days | 21.04 ± 16.12 | 18.86 ± 14.14 | 23.21 ± 17.66 | 4.35 (1.35, 7.34) | 0.005 |

**Abbreviations:** OR, odds ratio; MD, mean difference; CI, confidence interval.

^a^ Effect sizes for all categorical variables are presented as ORs with their 95% CIs, while the continuous variable Hospital stay is expressed as MD with its 95% CI.

^b^ Favorable outcome was defined as a mRS score of 0-2.

**eTable 4. E-value for the effect of Pre-admission statin on the primary and secondary outcomes**

| **Outcome** | **Effect estimate**  **(95% CI) ^a^** | ***P* value** | **E-value ^b^** | **E-value for CI ^b^** |
| --- | --- | --- | --- | --- |
| **Primary** |  |  |  |  |
| Favorable outcome at 30 days, n (%) ^c^ | 5.00 (3.00-8.32) | <0.001 | 3.89 | 2.86 |
| **Secondary outcome** |  |  |  |  |
| Favorable outcome at 90 days, n (%) ^c^ | 1.44 (0.94-2.23) |  | 1.69 | 1.00 |
| All-cause mortality at 90 days, n (%) | 0.70 (0.31-1.59) | 0.392 | 2.21 | 1.00 |
| Recurrent cerebral hemorrhage, n (%) | 1.52 (0.46-5.07) | 0.492 | 2.41 | 1.00 |
| Hydrocephalus, n (%) | 0.75 (0.49-1.16) | 0.199 | 2.00 | 1.00 |
| External ventricular drainage, n (%) | 0.26 (0.12-0.54) | <0.001 | 7.15 | 3.11 |
| Seizure, n (%) | 0.84 (0.12-6.05) | 0.864 | 1.67 | 1.00 |
| Neutrophil elevation, n (%) | 1.72 (1.09-2.97) | 0.019 | 2.83 | 1.40 |
| Transaminase elevation, n (%) | 3.44 (1.82-6.50) | <0.001 | 6.34 | 3.04 |
| Hospital stay, mean ± SD, days | 3.73 (0.84, 6.62) | 0.005 | 59.1 | 3.78 |

^a^ Adjusted for age, gender, systolic blood pressure, glucose, LDL, smoking, alcohol consumption, medical histories of hypertension, diabetes, heart disease, and stroke, prior use of antithrombotic agents, and WFNS grade. Effect sizes for all categorical variables are presented as ORs with their 95% CIs, while the continuous variable Hospital stay is expressed as MD with its 95% CI.

^b^ The E-value is defined as the minimum strength of association on the risk ratio scale that that an unmeasured confounder would need to have with both the exposure and the outcome, conditional on the measured covariates, to fully explain away a specific exposure-outcome association.

^c^ Favorable outcome was defined as a mRS score of 0-2.
